# Potential role of mitochondria and endoplasmic reticulum in the response elicited by D-aspartate in TM4 Sertoli cells

**DOI:** 10.3389/fcell.2024.1438231

**Published:** 2024-07-22

**Authors:** Sara Falvo, Giulia Grillo, Debora Latino, Gabriella Chieffi Baccari, Maria Maddalena Di Fiore, Massimo Venditti, Giuseppe Petito, Alessandra Santillo

**Affiliations:** ^1^ Department of Environmental Biological and Pharmaceutical Sciences and Technologies, University of Campania Luigi Vanvitelli, Caserta, Italy; ^2^ Department of Experimental Medicine, University of Campania Luigi Vanvitelli, Naples, Italy

**Keywords:** D-aspartate, Sertoli cells, mitochondrial dynamics, endoplasmic reticulum stress, MAM

## Abstract

D-Aspartic Acid (D-Asp) affects spermatogenesis by enhancing the biosynthesis of the sex steroid hormones acting either through the hypothalamus-pituitary–testis axis or directly on Leydig cells. Recently, *in vitro* studies have also demonstrated the direct effects of D-Asp on the proliferation and/or activity of germ cells. However, although D-Asp is present in Sertoli cells (SC), the specific role of the amino acid in these cells remains unknown. This study investigated the effects of D-Asp on the proliferation and activity of TM4 SC, focusing on the mitochondrial compartment and its association with the endoplasmic reticulum (ER). We found that D-Asp enhanced the proliferation and activity of TM4 cells as evidenced by the activation of ERK/Akt/PCNA pathway and the increase in the protein levels of the androgen receptor. Furthermore, D-Asp reduced both the oxidative stress and apoptotic process. An increase in mitochondrial functionality and dynamics, as well as a reduction in ER stress, were also found in D-Asp-treated TM4 cells. It is known that mitochondria are closely associated with ER to form the Mitochondrial-Associated Endoplasmic Reticulum Membranes (MAM), the site of calcium ions and lipid transfer from ER to the mitochondria, and *vice versa*. The data demonstrated that D-Asp induced stabilization of MAM in TM4 cells. In conclusion, this study is the first to demonstrate a direct effect of D-Asp on SC activity and to clarify the cellular/molecular mechanism underlying these effects, suggesting that D-Asp could stimulate spermatogenesis by improving the efficiency of SC.

## Introduction

Steroidogenesis and spermatogenesis appear to be modulated by a diversity of hormones and intracellular signaling pathways, which in turn are under control of a variety of factors (Flück and Pandey, 2014; Smith and Walker, 2014; Tremblay, 2015). Among these, D-aspartate (D-Asp), an amino acid endogenously present in testes of vertebrates, has received much attention (Burrone et al., 2010; Di Giovanni et al., 2010; Di Fiore et al., 2014; Di Fiore, Burrone et al., 2016; Di Fiore, Santillo et al., 2016; Chieffi Baccari et al., 2020; Usiello et al., 2020). Specifically, it is present in the Leydig cells (LC), Sertoli cells (SC), and germ cells (GC) of rodent and human testes (Sakai et al., 1998; D’Aniello et al., 2005; Tomita et al., 2016).

Intraperitoneal and oral administration of D-Asp to adult rats resulted in its accumulation in the testis and induced an increase in the levels of serum luteinizing hormone, progesterone, and testis/serum testosterone (D’Aniello et al., 2000; Santillo et al., 2014; Latino et al., 2024), acting either on the hypothalamus-pituitary–testis axis (D’Aniello et al., 2000) or directly on LC (Nagata, Homma, Lee et al., 1999; Nagata, Homma, Matsumoto et al., 1999). The evidence for the direct action of D-Asp on steroidogenesis/spermatogenesis emerged from several *in vitro* studies (Nagata, Homma, Lee et al., 1999; Nagata, Homma, Matsumoto et al., 1999; Raucci et al., 2014; Di Nisio et al., 2016; Santillo et al., 2016; Santillo, Falvo et al., 2019; Falvo et al., 2022). Specifically, in rat LC, D-Asp, alone or in the presence of human chorionic gonadotropin, stimulated the synthesis of testosterone by stimulating the gene/protein expression of the steroidogenic acute regulatory protein (StAR), a transport protein that regulates cholesterol transfer within the mitochondria (Nagata, Homma, Lee et al., 1999; Nagata, Homma, Matsumoto et al., 1999; Raucci et al., 2014; Di Nisio et al., 2016). Studies conducted on murine spermatogonia (SPG) (GC-1), and spermatocyte (SPC) (GC-2) cell lines have also shown that D-Asp induces phosphorylation of the ERK and Akt, and upregulates the expressions of mitotic and meiotic markers, respectively (Santillo et al., 2016; Santillo, Falvo et al., 2019; Santillo, Venditti et al., 2019; Falvo et al., 2022). Furthermore, a direct role of D-Asp in the capacitation process and acrosome reaction has been demonstrated in spermatozoa from young mice (Raspa et al., 2022). Moreover, the addition of a commercial mixture comprising D-Asp, Coenzyme Q10, and zinc (CZA) to the culture medium diluting the spermatozoa of sub-fertile patients prevented the decline in sperm kinetics, and protected the spermatozoa from DNA fragmentation and lipid peroxidation, particularly in the oligospermic samples (D’Aniello et al., 2005; Giacone et al., 2017).

Spermatogenesis and sperm motility require energy continuously; therefore, mitochondria, being the main energy producers in cells, play a key role in these processes. In this regard, it has recently been demonstrated that *in vivo* D-Asp administration activates mitochondrial biogenesis and mitochondrial dynamics in both GC and LC (Falvo et al., 2022; Latino et al., 2024).

It is known that 5%–20% of the mitochondria interacts to the endoplasmic reticulum (ER) to form the Mitochondrial-Associated Endoplasmic Reticulum Membranes (MAM) (Rizzuto et al., 1998; Ni and Yuan, 2021) and that the structure of MAM changes in relationship to different cell states. MAM is involved in the transport of calcium between ER and mitochondria, cell proliferation, cell apoptosis, and other cell physiological or pathological processes (Filadi et al., 2017; Krzysko et al., 2022). Interestingly, in LC D-Asp improves the mitochondria- ER association, which is of crucial importance for efficient steroidogenesis (Latino et al., 2024).

However, although D-Asp is present in SC, the specific role of the amino acid in these cells remains unknown. SC are testicular somatic cells and perform crucial functions in gametogenesis; they have important “nursing” functions for spermatogenic cells and provide physical support and energy sources to GCs as well as nutrients necessary for testis development and spermatogenesis (Galardo et al., 2014; O’ Donnell et al., 2022). In the developing testis, SC differentiation coordinates the differentiation of other somatic cells, including LC, and of the GC (Makela et al., 2019). Normal differentiation and maintenance of SC identity are decisive for the future reproductive health, and their number correlates with testicular volume and determines the number of spermatozoa produced per day (Makela et al., 2019).

Therefore, a better knowledge about the role of factors involved in the regulation of SC homeostasis and functions is important for the male reproductive function (Lucas et al., 2014). For this purpose, we investigated the effects of D-Asp on the proliferation and activity of cultured mouse Sertoli (TM4) cells, focusing on the mitochondrial compartment and its association with the ER, to study the cellular/molecular mechanisms stimulated by D-Asp in these cells.

## Materials and methods

### Cell culture and treatments

TM4 mouse cell line (Sertoli cells, ATCC CRL-1715; Washington, United States) was cultured in Dulbecco’s modified Eagle’s Medium F/12 (DMEM F/12), supplemented with 5% horse serum and 2.5% Fetal Bovine Serum (FBS), and grown in a 37°C humidified atmosphere of 5% CO_2_ (Grillo et al., 2024). Cells were treated with D-aspartate 100 µM diluted in distilled H_2_O (Sigma Aldrich, Milan, Italy) at different exposure times (30 min, 2 h and 4 h). The dose was chosen based on preliminary experimental studies. Control cultures were treated with the vehicle alone. The experiment was performed in triplicate.

### Protein extraction and western blot analysis

The total protein cell extracts were obtained as described in Grillo et al. (2024). Briefly, the samples were homogenized in the lysis buffer containing 50 mM Tris-HCl (pH 7.5), 5 mM EDTA, 300 mM NaCl, 150 mM KCl, 1 mM dithiothreitol, 1% Nonidet P40, and a mix of protease inhibitors (all from Sigma Aldrich, Milan, Italy). After centrifugation at 12,000 *g* for 10 min both cytoplasmic and nuclear soluble proteins resulted in the supernatants. Protein concentration in the extracts was determined by the Bradford assay (Bio-Rad, Melville, NY, United States). Then, protein extracts were separated on a sodium dodecyl sulfate-polyacrylamide gel electrophoresis (SDS-PAGE) gel and then transferred to a nitrocellulose membrane (Bio-Rad, Melville, NY, United States). Next, the membranes were treated with a blocking solution for 1 h and then incubated overnight at 4°C with the respective primary antibodies (refer to Supplementary Table S1 for the list of the used primary antibodies). The reaction bands were detected and quantified as described in Venditti et al. (2020).

### Lipid peroxidation assay

The effect of D-Asp on TM4 cells’ oxidative damage for lipids was detected by a quantitative analysis of malondialdehyde (MDA) using a commercial kit (#ab118970; Abcam, Cambridge, United Kingdom). MDA content was expressed as nmol/mg protein.

### DAPI staining

To evaluate the apoptotic cells, 5 × 10^3^ TM4 cells were plated on a coverslip, stained with DAPI solution. Apoptotic cells show characteristic changes in their nuclei, such as chromatin condensation, DNA fragmentation and formation of apoptotic bodies (Atale et al., 2014). The intensity of staining of DAPI, a fluorescent dye that stain the nuclei, increases when the chromatin in nuclei is condensed, highlighting apoptotic cells (Dmitrieva and Burg, 2008). DAPI-stained cells were observed under an optical microscope (Leica DM 5000 B + CTR 5000; Leica Microsystems, Wetzlar, Germany) with UV lamp. A Leica DFC320 R2 digital camera was used to take the photographs. The images were analyzed and saved with IM 1000 software (version 4.7.0; Leica Microsystems, Wetzlar, Germany).

### Mitochondrial membrane potential (MMP) assay

MMP assay in D-Asp-treated TM4 cells was detected by a commercial assay kit (#ab113852; Abcam, Cambridge, United Kingdom). 5 × 10^3^ cells were plated for the assay and TMRE positive cells were observed under a fluorescence microscope (Leica DMLB; Leica Microsystems, Wetzlar, Germany).

### Statistical analysis

To evaluate significant changes among experimental groups an ANOVA followed by a Student- Newman-Keuls test was used. Values for *p* < 0.05 were considered statistically significant. All data were expressed as the mean ± S.D. of three separate experiments.

## Results

### Effects of D-Asp on TM4 cell proliferation

**FIGURE 1:**
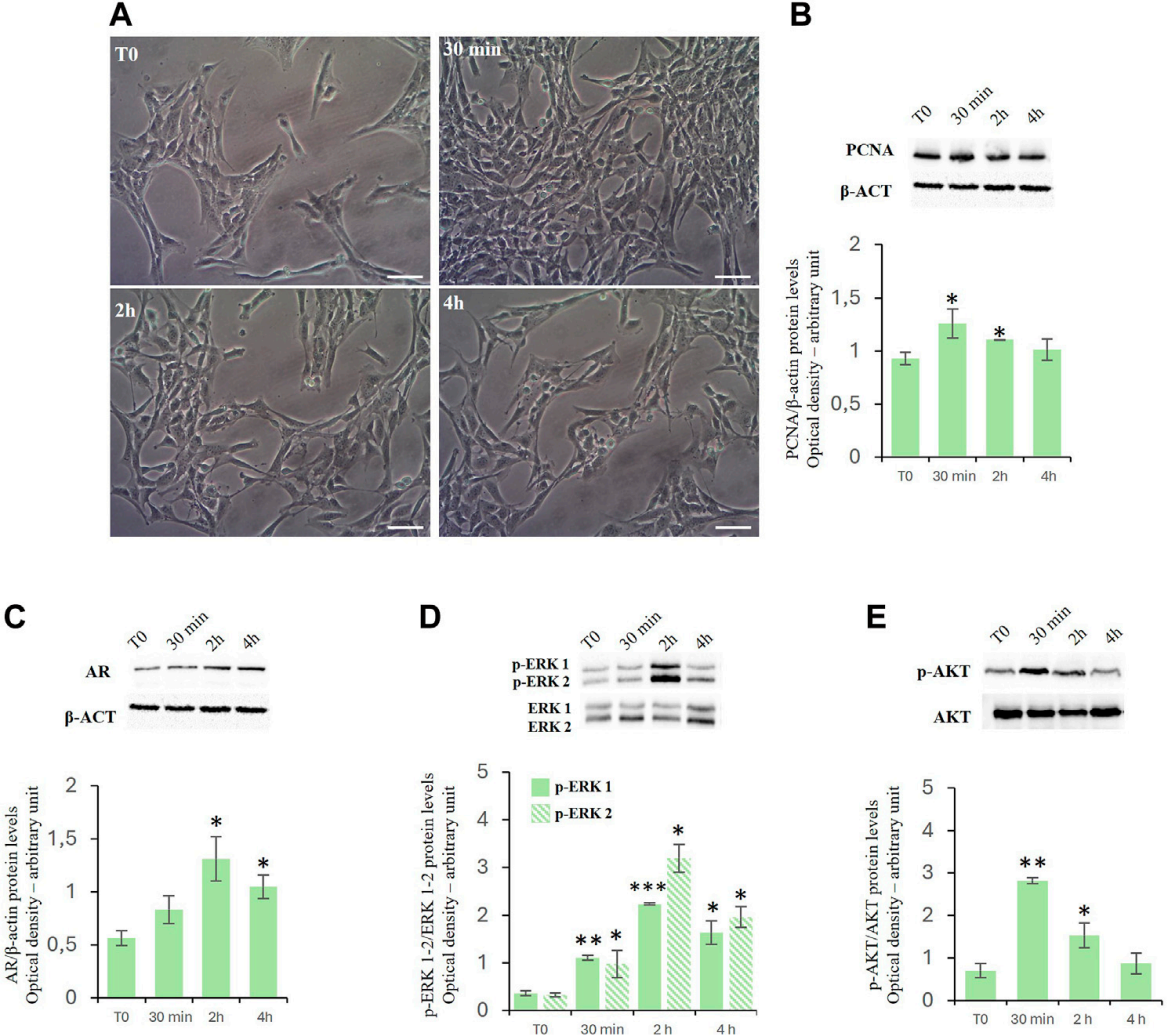
**(A,B)** D-Asp affects TM4 cell proliferation. **(A)** TM4 cell growth after 30 min, 2 and 4 h after D-Asp treatment. Images were captured at ×20 magnification. Scale bars represent 20 µm. **(B)** PCNA (36 kDa) expression levels were detected by western blot analysis in TM4 from control and D-Asp treated cells. **(C–E)** Expression of androgen receptor and ERK/Akt signaling. **(C)** AR (110 kDa), **(D)** phospho-ERK1/2 (42–44 kDa) and **(E)** phospho-Akt (60 kDa) protein levels were detected by western blot in TM4 from control and D-Asp treated cells. Protein levels were quantified using ImageJ program. PCNA and AR were normalized with the respect to β-actin (42 kDa); p-ERK 1/2 and p-Akt were normalized with respect to total ERK1/2 and Akt, respectively. Data represent the mean ± standard deviation of three different experiments. *P < 0.05, **P < 0.01 and ***P < 0.001.

Treatment with D-Asp increased the TM4 cell density at 30 min and 2 h of exposure compared to control (Figure 1A). These findings were supported by the biochemical analysis (Figure 1B). We found that treatment with D-Asp induced an increase in the levels of PCNA, a protein expressed in the nucleus of cells in the S phase and used as mitotic marker, by 35% at 30 min and 18% at 2 h compared to control cells; at 4 h the values returned to control levels (Figure 1B).

### D-asp upregulated androgen receptor protein levels and ERK/Akt signaling

In TM4 cells, D-Asp induced a significant increase in the expression of the androgen receptor (AR) at 2 and 4 h (Figure 1C).

Further we have investigated the role exerted by D-Asp on the activities of ERK, a signaling protein belonging to the Mitogen-activated protein kinases (Zhang et al., 1995; Cobb, 1999), and Akt, key protein in regulating the cell cycle (Vogiatzi and Giordano, 2007). In D-Asp-treated TM4 cells, both ERK1 and ERK2 activities (p-ERK1 and p-ERK2) increased at 30 min, and remained high until 4 h (Figure 1D). Furthermore, D-Asp treatment induced a significant increase of p-Akt after 30 min and 2 h of incubation; p-Akt levels returned to the baseline after 4 h (Figure 1E).

### Effects of D-Asp on cellular oxidation levels

**FIGURE 2:**
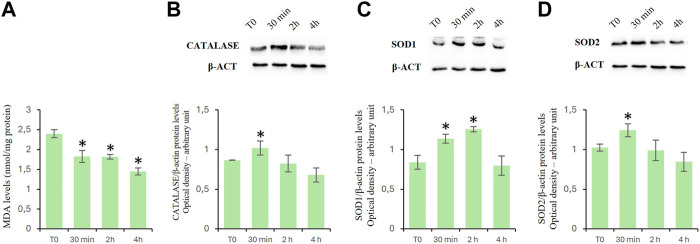
Oxidation levels in TM4 cells after D-Asp treatment. **(A)** MDA amount in control and D-Asp treated cells. **(B–D)** Western blot detection of **(B)** catalase (60 kDa), **(C)** SOD1 (23 kDa) and **(D)** SOD2 (22 kDa) in TM4 cell lysates. Protein levels were quantified by ImageJ program and normalized *versus* β-actin (42 kDa). Data represent the mean ± standard deviation of three separate experiments. *P < 0.05.

To assay the effects of D-Asp on oxidative stress in TM4 cells, we evaluated the MDA levels as well as the expression levels of catalase, SOD1 and mitochondrial SOD2 proteins (Figure 2).

MDA is the main by-product formed during lipid peroxidation (Ayala et al., 2014). D-Asp treatment significantly reduced the MDA levels in a time-manner dependent (Figure 2A).

Catalase, SOD1, and SOD2 are antioxidant enzymes. In particular, catalase catalyzes the decomposition of hydrogen peroxide into water and oxygen (Salvi et al., 2007); superoxide dismutase 1 (SOD1) catalyzes the dismutation reaction of the superoxide radical into hydrogen peroxide and molecular oxygen in the cytoplasm and SOD2 in the mitochondria (Abreu and Cabelli, 2010). In D-Asp-treated TM4 cells, the protein levels of catalase (Figure 2B), SOD1 (Figure 2C) and SOD2 (Figure 2D) significantly enhanced at 30 min, and only SOD1 remained high at 2 h (Figure 2C); at 4 h the values returned next to T0 (Figures 2B–D).

### D-Asp reduced the apoptosis

**FIGURE 3:**
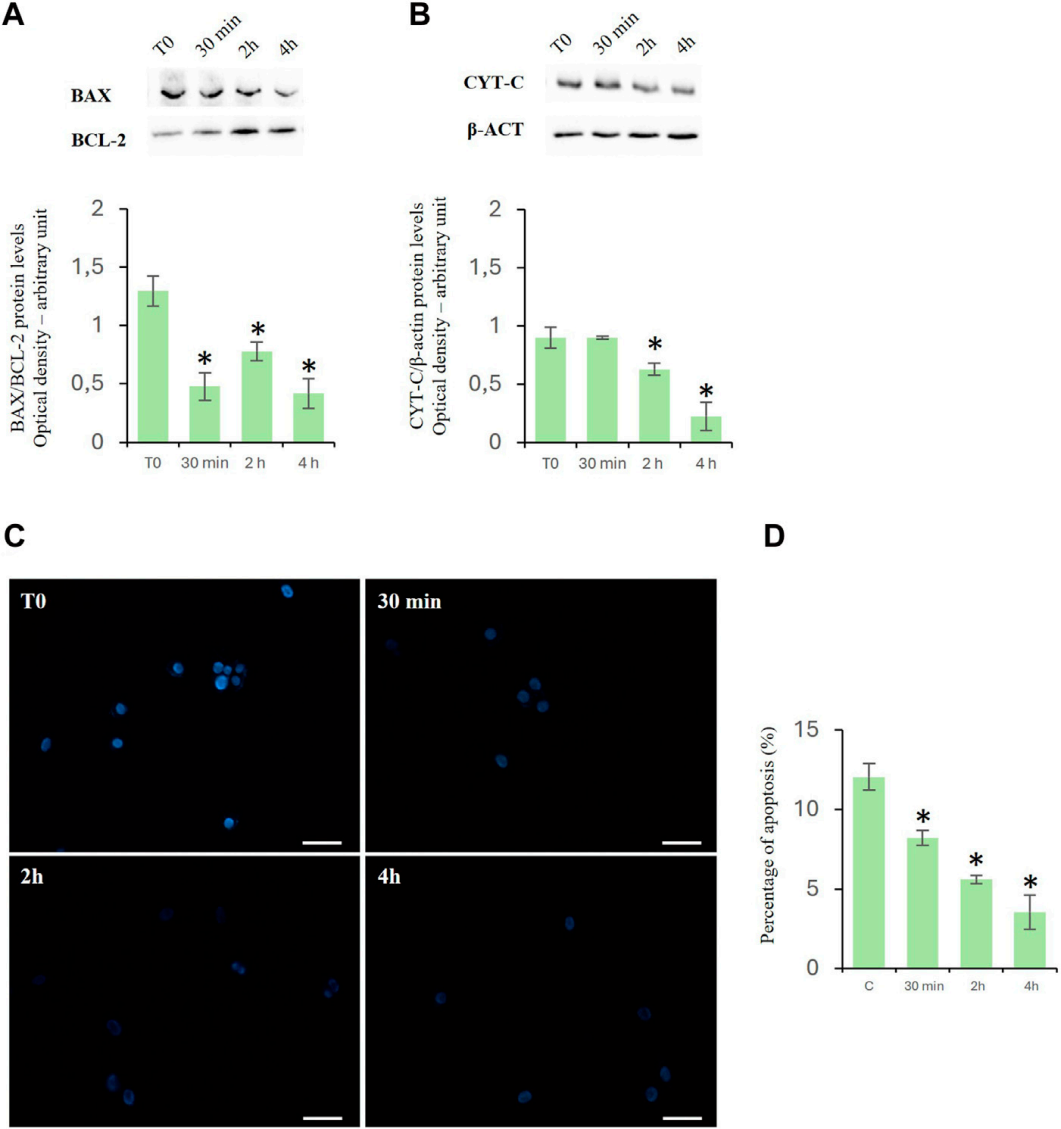
Effects of D-Asp on apoptosis in TM4 cells. **(A)** Bax (23 kDa) and Bcl-2 (25 kDa), and **(B)** cyt c (14 kDa) protein levels were detected by western blot analysis in control and D-Asp-treated TM4 cells. Histograms show the Bax/Bcl-2 ratio and cyt c relative protein levels, respectively. Protein levels were quantified by ImageJ program and normalized with the respect to β-actin (42 kDa). Data represent the mean ± standard deviation of three different experiments. *P < 0.05. **(C)** DAPI staining of TM4 cells after D-Asp treatment. Images were captured at ×40 magnification. Scale bars represent 40 µm. **(D)** Histogram shows the percentage of DAPI-positive nuclei. Data represent the mean ± standard deviation of three experiments. *P < 0.05 vs. control.

Cytochrome c is a prominent marker of apoptosis and of Bax/Bcl2 ratio represents an apoptotic index used to evaluate the balance between apoptotic and anti-apoptotic proteins. Treatment with D-Asp significantly reduced the ratio of Bax/Bcl-2 protein levels in the TM4 cells at all examined times (Figure 3A). In the presence of D-Asp, cytochrome c protein was significantly decreased at 2 and 4 h (Figure 3B).

The above findings were confirmed by the DAPI staining, which was used to detect apoptotic cells by evidencing the chromatin condensation and morphological changes of nuclei. The results showed a time-dependent decrease in the percentage of DAPI-positive nuclei in D-Asp-treated TM4 cells, compared to that of the control group, suggesting that D-Asp significantly decreased the percentage of apoptotic cells (Figures 3C, D).

### Effects of D-Asp on mitochondrial functionality

As mitochondria perform various functions, such as ATP synthesis, intrinsic apoptosis, and calcium signaling, we investigated the mitochondrial membrane potential (MMP) and oxidative phosphorylation system (OXPHOS), to assess mitochondrial functionality.

**FIGURE 4:**
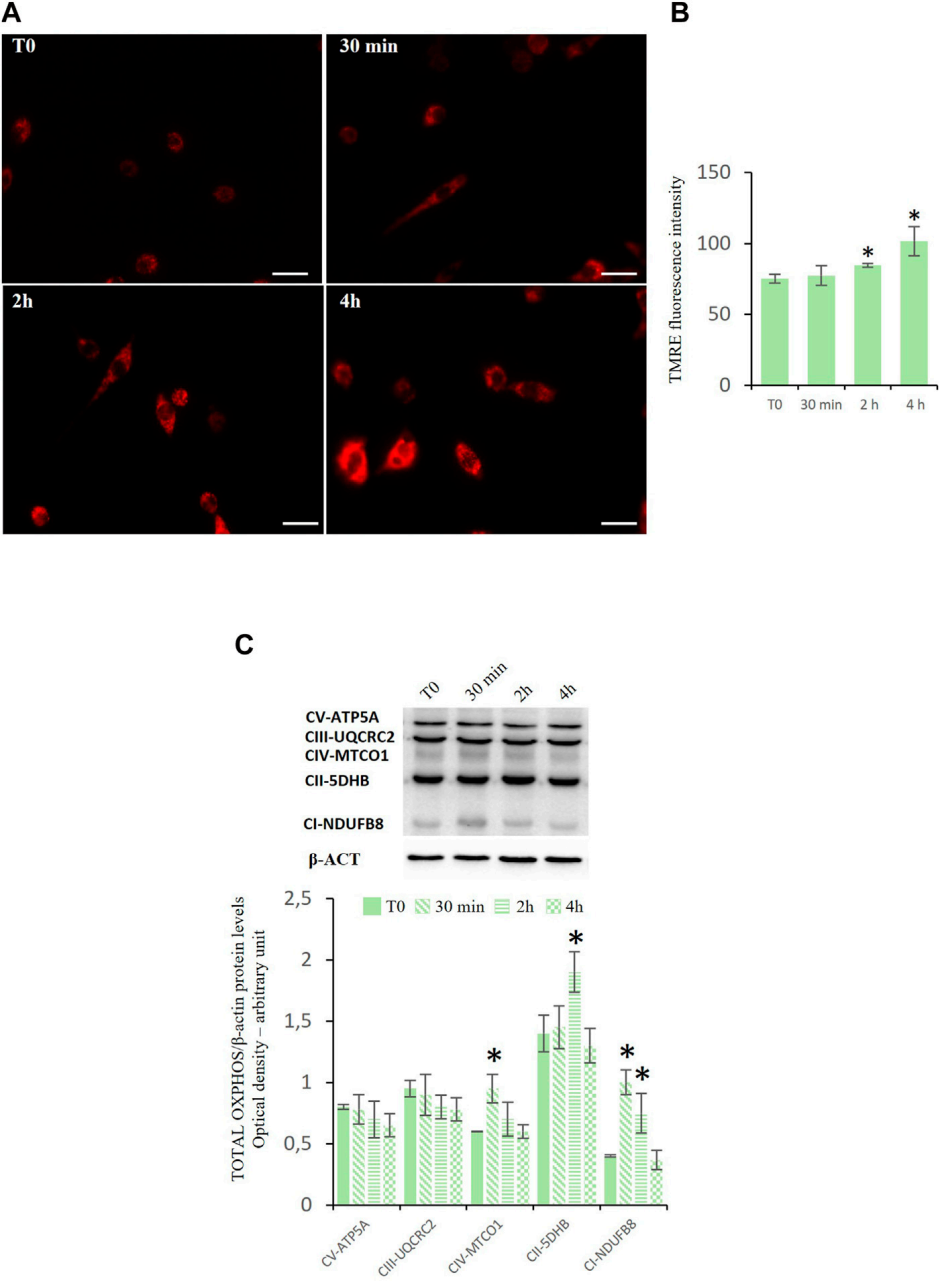
Mitochondrial functions in TM4 D-Asp treated cells. **(A)** Determination of MMP in TM4 cells through detecting TMRE-positive cells (red). The images were captured at ×40 magnification under fluorescence microscope. Scale bars represent 40 µm. **(B)** The histogram shows TMRE fluorescence intensity that was analyzed by ImageJ software. **(C)** Western blot detection of Oxidative Phosphorylation System (OXPHOS) complexes in TM4 cells at various times of D-Asp treatment. The amounts of proteins were quantified by ImageJ program and normalized with *versus* β-actin. The figure shows the complexes in order of their appearance in Western blotting analysis (molecular weight). Data represent the mean ± standard deviation of three separate experiments. *P < 0.05 *versus* controls.

To evaluate the mitochondrial potential membrane (MMP) we used the staining with TMRE that is a cell permeant, positively charged, red-orange dye that readily accumulates in active mitochondria. The results indicated that D-Asp increased the percentage of TMRE-positive TM4 cells at 2 and 4 h (Figures 4A, B).

Figure 4C showed the expression levels of enzymes representing OXPHOS complexes in the TM4 cell lysates after the incubation with D-Asp. The OXPHOS complexes, present on the mitochondrial inner membrane, are composed of several enzymes, including NADH: ubiquinone oxidoreductase (complex I, CI-NDUFB8), succinate dehydrogenase (complex II, CII-5DHB), ubiquinol-cytochrome c oxidoreductase (complex III, CIII-UQCRC2), cytochrome c oxidase (complex IV, CIV-MTCO1), and ATP synthase (complex V, CV-ATP5A). These protein complexes (complex I–IV) are responsible for a series of reversible oxidation and reduction reactions of the nicotinamide adenine dinucleotide (NAD^+^/NADH) or flavine-adenine dinucleotide (FAD^+^/FADH_2_), which generate a proton gradient across the intermembrane space in the mitochondria. The proton gradient is utilized by the complex V (ATP synthase) to synthetize ATP from ADP and organic phosphate. D-Asp increased the expression levels of the complexes CIV-MTCO1 and CII-5DHB at 30 min and 2 h, respectively (Figure 4C); the complex CI-NDUFB8 was upregulated at 30 min and 2 h after D-Asp treatment (Figure 4C). The complexes CIII-UQCRC2 and CV-ATP5A in the TM4 cells remained unchanged after the D-Asp treatment (Figure 4C).

### Effects of D-Asp on the protein expression of mitochondrial biogenesis-, fusion- and fission-related factors

**FIGURE 5:**
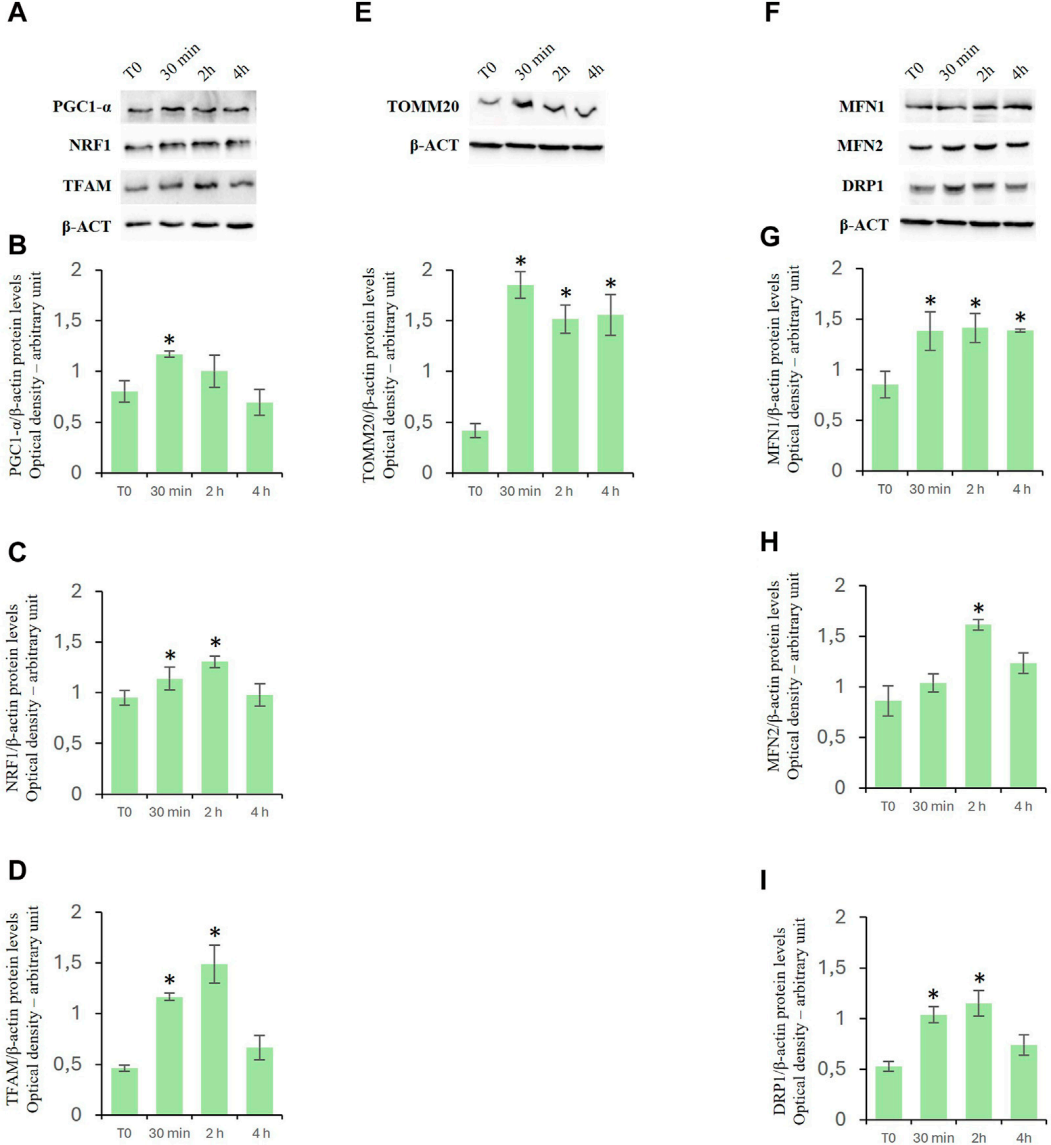
Analysis of mitochondrial biogenesis-, fusion- and fission-related factors in TM4 cells after D-Asp treatment. Western blot detection of **(A–D)** PGC-1α (110 kDa), NRF1 (68 kDa) and TFAM (28 kDa), **(E)** TOMM20 (20 kDa) and **(F–I)** MFN1 (84 kDa), MFN2 (86 kDa), and DRP1 (78–82 kDa) protein levels in TM4 from control and D-Asp cell-treated. Protein levels were quantified using the ImageJ program and normalized with the respective to β-actin (42 kDa). Data represent the mean ± standard deviation of three separate experiments. *P < 0.05.

To investigate the effects of D-Asp on mitochondrial biogenesis, specific markers such as peroxisome proliferative activated receptor gamma coactivator 1α (PGC-1α), nuclear respiratory factor 1 (NRF1), and mitochondrial transcription factor A (TFAM) were employed (Viña et al., 2009; Hees and Harbauer, 2022). PGC-1α is a positive regulator of mitochondrial biogenesis and respiration and is also reported to play a central role in the detoxification of ROS. NRF1 upregulates the expression of nuclear genes encoding TFAM and other mitochondrial proteins. TFAM is then transported into mitochondria where it binds to mitochondrial DNA (mtDNA) and activates the transcription and replication of the mtDNA essential for the generation of new mitochondria. D-Asp treatment significantly increased the protein levels of PGC-1α in the TM4 cells at 30 min of incubation (Figures 5A, B). Protein levels of NRF1 and TFAM were significantly enhanced at 30 min and 2 h after D-Asp addition (Figures 5A, C, D). At 4 h PGC-1α, NRF1, and TFAM protein levels returned to the baseline (Figures 5A–D).

Expression levels of TOMM20, a peripheral component of the TOM complex that acts as a primary receptor for mitochondrial precursor proteins (Hachiya et al., 1995), were evaluated to determine the mitochondrial mass. In D-Asp-treated cells, TOMM20 expression levels were significantly higher at all the evaluated incubation times (30 min–4 h) compared to that in untreated cells (Figure 5E).

To assay the effects of D-Asp on mitochondrial fusion and fission, Mitofusin (MFN1 and MFN2) and Dynamin-Related Protein 1 (DRP1) were employed as specific markers, respectively (Lee and Yoon, 2016; Tilokani et al., 2018). Particularly, Mitofusin (MFN1 and MFN2) mediate the outer mitochondrial membrane fusion and DRP1 is a key enzyme for mitochondrial fission as it forms the helical oligomers that envelop the outer mitochondrial membrane and then cleave it. The D-Asp-treated TM4 cells exhibited a significant increase in the MFN1 protein expression (Figures 5F, G) at all the evaluated incubation times (30 min–4 h) and at 2 h in MFN2 (Figures 5F, H) upon the addition of D-Asp. The expression of the DRP1 protein was observed to significantly increase at 30 min and 2 h after the addition of D-Asp (Figures 5F, I).

### D-Asp increased expression levels of MAM-related proteins

**FIGURE 6:**
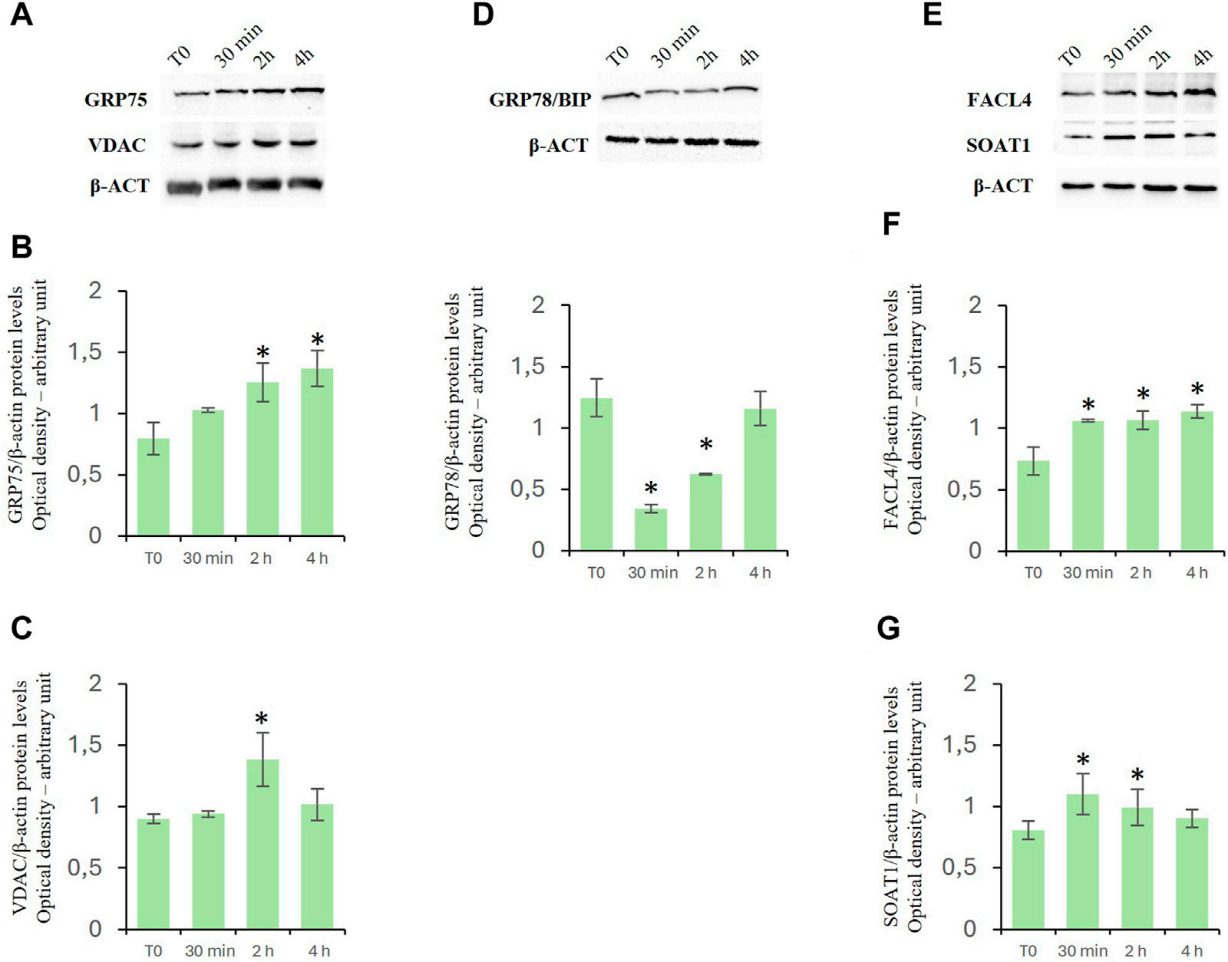
Effects of D-Asp on Mitochondrial-Associated Endoplasmic Reticulum Membrane (MAM) in TM4 cell line. Western blot analysis of **(A–C)** GRP75 (75 kDa) and VDAC (32 kDa), **(D)** GRP78/BIP (78 kDa), **(E–G)** FACL4 (79 kDa) and SOAT1 (47 kDa) protein levels in TM4 from control and D-Asp cell-treated. Protein levels were quantified by ImageJ program and normalized *versus* β-actin (42 kDa). Data represent the mean ± standard deviation of three different experiments. *P < 0.05 vs. control.

To investigate the effects of D-Asp on mitochondrial-endoplasmic reticulum association in TM4 cells, we investigated the levels of expression of both glucose-regulated protein 75 (GRP75) (Figures 6A, B) and voltage-dependent anion channel (VDAC) (Figures 6A, C) proteins. GRP75 and VDAC are localized on MAMs and regulate Ca2+ release from ER for its efficient mitochondrial uptake (Szymanski et al., 2017). D-Asp significantly increased GRP75 and VDAC protein levels at 2 h after the treatment (Figures 6A, B); at 4 h, GRP75 remained still high, whereas VDAC returned to basal levels (Figures 6A, C).

Furthermore, the protein levels of binding immunoglobulin protein (GRP78/Bip), a useful marker to evaluate ER stress. This protein is a heat shock protein that acts as an important chaperone facilitating protein folding, and preventing the accumulation of protein aggregates in the ER (Lee, 2005). GRP78/Bip decreased in D-Asp treated TM4 cells at 30 min and 2 h (Figure 6D); it was comparable to T0 level after 4 h from D-Asp treatment (Figure 6D).

Finally, we investigated the expression levels of fatty acid CoA ligase 4 (FACL4), a key enzyme required for the synthesis of phospholipids, and Sterol O-Acyltransferase 1 (SOAT1), which converts free cholesterol to cholesterol ester for its storage (Latino et al., 2024). Both FACL4 (Figures 6E, F) and SOAT1 (Figures 6E, G) protein levels significantly increased in D-Asp-treated TM4 cells at 30 min and 2 h; at 4 h, FACL4 remained still high, whereas SOAT1 returned to basal levels (Figure 6E–G).

## Discussion

D-Asp is known to affect spermatogenesis by promoting the biosynthesis of the sex steroid hormones acting either through the hypothalamus-pituitary–testis axis (D’Aniello et al., 2000; Santillo et al., 2023) or by directly on LC (Nagata et al., 1999; Nagata et al., 1999). Furthermore, *in vitro* studies have demonstrated the direct effects of D-Asp on the proliferation and/or activity of GC (Santillo et al., 2016; Santillo et al., 2019; Giacone et al., 2017; Falvo et al., 2022). A study on the effects of D-Asp on SCs, somatic cells playing an essential role in maintaining testicular functions, remains still missing. Our study aimed to investigate the cellular response induced by D-Asp in TM4 cells, a non-tumorigenic Sertoli cell line isolated from the testis of 11–13 old-days mice. TM4 cells share many characteristics of Sertoli cells, such as expression of follicle stimulating hormone (FSH), androgen receptor, progesterone receptor (Mather, 1980). These cells have been widely used as a substitute for primary SCs and can be considered as a valid model to draw physiological conclusions (Li et al., 2022; Grillo et al., 2024). In this regard, it has been showed that diosgenin treatment increased cell viability and proliferation in both TM4 cells and primary SCs in a dose-dependent manner (Wu et al., 2015) and, both Dibutyl/Monobutyl phthalate (Ma et al., 2020) and Bisphenol A (Ge et al., 2014) stimulated the proliferation of TM4 at a relatively low concentration range. Noteworthy, the timing of action of D-Asp on SC, prevalently at 30 min and 2 h, confirmed that this amino acid can rapidly promote testicular activity (Di Fiore et al., 2019). The results revealed, for the first time, that D-Asp stimulated the ERK/Akt activity in the TM4 and induced the expression of PCNA. The ERK/Akt pathway is well-recognized to be involved in the regulation of several biological functions, including proliferation and apoptosis (Coffer et al., 1998; Vogiatzi and Giordano, 2007). In particular, ERK and Akt pathways were reported to play a crucial role in GC proliferation (Sette et al., 1999; Hasegawa et al., 2013; Santillo et al., 2014, 2016; Santillo et al., 2019; Santillo et al., 2019; Falvo et al., 2022). Therefore, in the present study, the increased levels of PCNA in the D-Asp-treated TM4 cells strongly suggested that this amino acid played a role in promoting the proliferative process in these cells; this hypothesis was confirmed by the morphological analysis. Several studies have shown that SC also divide in adult animals, including mice, hamsters, and humans, under experimental and physiological conditions (Lucas et al., 2014; O’Donnell et al., 2022). Furthermore, present findings agreed with those of our previous studies, demonstrating that D-Asp upregulated the PCNA expression via the ERK/Akt pathway in both GC-1 (Santillo et al., 2016) and GC-2 cells (Falvo et al., 2022) as well as in rat testis (Venditti et al., 2020). Furthermore, we found that D-Asp enhanced the protein expression of AR in TM4 cells. AR is known to play a key role in male reproductive physiology and participates in the regulation of SC proliferation by cross-talking with ERK and Akt signaling pathway (Cheng et al., 2007; Willems et al., 2010; Yang et al., 2021; Wang et al., 2022). Specifically, in Sertoli cells testosterone induces AR migration to the plasma membrane and AR-Src interaction; in turn, Src kinase activation is required for stimulation of ERK, that plays an essential role in many cellular processes, including cell proliferation (Cheng et al., 2007). Consistently, the AR suppression inhibits the TM4 cells proliferation by negatively regulating Akt pathway (Wang et al., 2022). Therefore, we hypothesized that D-Asp could induce SC proliferation by activating the AR-mediated ERK/Akt signaling pathway, thus promoting spermatogenesis.

In addition, we found for the first time that D-Asp was able to specifically reduce oxidative stress in TM4 cells, as evidenced by the decrease in the levels of MDA, the main by-product formed during lipid peroxidation, and the increase in the protein expression of antioxidant enzymes, such as catalase, SOD1, and SOD2. Therefore, oxidative stress reduction induced by D-Asp could favor SC proliferation. Our findings were in accordance with our previous *in vivo* studies demonstrating that D-Asp was able to counteract/prevent the oxidative stress induced by ethane dimethane sulfonate and cadmium in rat testis (Venditti et al., 2021, 2023; Latino et al., 2024). Consistently, Giacone et al. (2017) reported that D-Asp reduced sperm lipid peroxidation in both normozospermic and asthenozoospermic men.

Herein, we found a decreased apoptosis in D-Asp-treated TM4 cells, as demonstrated by the decrease in the cytochrome c and ratio of the Bax/Bcl2 protein levels, as well as, by the reduction of DAPI-positive nuclei percentage. Therefore, the abovementioned D-Asp-mediated activation of the ERK/Akt signaling pathway as well as the decrease in oxidative stress might explain the inhibition of the apoptosis process in TM4 cells.

Finally, to assess the cellular response induced by D-Asp in TM4 cells, we evaluated the functionality and dynamics of the mitochondrial compartment and its association with MAM-mediated ER.

The mitochondrion is the central system for energy production, and it is intimately related to male reproduction by modulating metabolism and apoptosis of testicular cells (Vertika et al., 2020; Zhang et al., 2022); a decrease in the MMP can affect ATP production with consequent damage or dysfunction of mitochondria (Amaral et al., 2013; Liu et al., 2022; Grillo et al., 2024). Our results revealed that D-Asp increased MMP in D-Asp-treated TM4 cells. Furthermore, we found an increase in the expression levels of some OXPHOS complex after D-Asp treatment. These protein complexes (complex I–IV) are responsible for a series of reversible oxidation and reduction reactions of the nicotinamide adenine dinucleotide (NAD^+^/NADH) or flavine-adenine dinucleotide (FAD^+^/FADH_2_), which generate a proton gradient across the intermembrane space in the mitochondria. The proton gradient is utilized by the complex V (ATP synthase) to synthetize ATP from ADP and organic phosphate. Our results showed that D-Asp significantly increased the expressions of the CI, CII, and CIV complexes, indicating that increased levels of oxidative phosphorylation in D-Asp-treated TM4 cells occurred. These findings confirmed the role of this amino acid in the activation of oxidative phosphorylation since we have previously found an increase in OXPHOS complexes in D-Asp-treated GC-2 cells (Falvo et al., 2022). Moreover, our results showed that D-Asp treatment triggered mitochondrial biogenesis in TM4 cells, as evidenced by an increase in the expression levels of PGC-1α, NRF-1, and TFAM proteins. In accord, an increase in the expression of TOMM20, a mitochondrial outer membrane receptor, indicated an increase in mitochondrial mass in TM4 after D-Asp treatment. Mitochondrial biogenesis and fusion/fission dynamics maintain shape, size, and number of mitochondria (Labbe et al., 2014). Alterations in mitochondrial dynamics impaired the functionality of both germ and somatic cells (Fang et al., 2018; Liu et al., 2022; Grillo et al., 2024). Here we observed that D-Asp treatment promoted mitochondrial dynamics, as evidenced by the increased expression level of MFN1 and MFN2 (fusion markers) and DRP1 (a fission marker). Our previous study demonstrated that D-Asp activated mitochondrial biogenesis and mitochondrial fusion in spermatocyte GC-2 cells, suggesting its participation in the metabolic shift occurring during meiosis and an active role for this amino acid in GC maturation (Falvo et al., 2022). An involvement of D-Asp in the functional improvement of mitochondria in LC has also been demonstrated (Latino et al., 2024).

The close coupling structure between mitochondria and ER facilitates material exchange and signal transmission between them (Gao et al., 2018; Perez-Leanos et al., 2021). The stabilization of the MAM structure contributes to cell homeostasis and depends on the functional proteins involved in the MAM (Luan et al., 2021). Consistently, it was demonstrated that in cells with knockdown of Mfn2, the MAM distance increased and the transfer of calcium from ER to mitochondria decreased, indicating that Mfn2 is essential for MAM structural stability (Das et al., 2022). In the present study, the increase in MNF2 expression suggested a stabilization in mitochondrion-endoplasmic reticulum association in D-Asp-treated TM4 cells. MAM plays a key role in the transport of calcium from the ER to mitochondria. Specifically, the inositol trisphosphate receptor (IP3R), located in the ER, interacts with VDAC, located in the mitochondrial outer membrane, via GRP75 (Ni and Yuan, 2021). Our results indicated that D-Asp treatment increased the levels of GRP75 and VDAC in TM4 cells; this could favor calcium transfer and reduce ER stress (Simmen et al., 2005). Interestingly, we found a decrease in the levels of GRP78/Bip, an ER chaperone in D-Asp-treated TM4 cells, thus indicating that ER stress signals were inhibited. The ER stress inhibition could be responsible, in turn, for MAM structure stabilization, and for the decreased apoptosis (Ma et al., 2023).

MAMs are also involved in lipid metabolism by participating in the transport of lipids between the ER and mitochondria. Protein levels of FACL4 and SOAT1, lipid-biosynthesis-involved enzymes, were significantly higher in D-Asp-treated TM4 cells compared to untreated cells. Accordingly, our previous *in vivo* study has evidenced that D-Asp affects steroidogenesis in rat LC by increasing the expression levels of FACL4 and SOAT1 proteins (Latino et al., 2024).

## Conclusion

To our knowledge, this is the first report of a direct effect of D-Asp on SC proliferation and activity. Here we showed the cellular/molecular mechanism underlying these effects. The data indicated that D-Asp induced proliferation in mouse TM4 cells through AR-mediated ERK/Akt/PCNA pathway and reduced oxidative stress and apoptosis. An increase in mitochondrial functionality and dynamics, stabilization of MAM, as well as a reduction in ER stress, were also found. Therefore, these results suggested that D-Asp could enhance spermatogenesis improving the SC efficiency. In conclusion, this study provided novel insights into understanding the mechanisms by which D-Asp exerts its role in spermatogenesis and male fertility.

## Data Availability

The original contributions presented in the study are included in the article/Supplementary Material, further inquiries can be directed to the corresponding author.

## Abbreviations

PCNA, Proliferating Cell Nuclear Antigen; AR, Androgen Receptor; ERK1/2, extracellular signal-regulated kinase 1/2; p-ERK1/2, phospho-extracellular signal-regulated kinase 1/2; AKT, protein kinase B; p-AKT, phospho-protein kinase B; SOD1, superoxide dismutase 1; SOD2, superoxide dismutase 2; BAX, Bcl-2–associated X protein; BCL-2, B-cell lymphoma; CYT C, cytochrome c; OXPHOS, oxidative phosphorylation complexes; CV-ATP5A, complex V-ATP synthase; CIV-MTCO1, complex IV-cytochrome c oxidase subunit 1; CIII-UQCRC2, complex III-ubiquinol-cytochrome c reductase core protein 2; CII-SDHB, complex II-succinate dehydrogenase iron-sulfur subunit; CI-NDUFB8, complex I-NADH:ubiquinone oxidoreductase subunit B8; PGC1-α, peroxisome proliferator-activated receptor-gamma coactivator -1alpha; NRF1, nuclear respiratory factor 1; TFAM, mitochondrial transcription factor A; TOMM20, translocase of the outer mitochondrial membrane complex subunit 20; MFN1, mitofusin 1; MFN2, mitofusin 2; DRP1, dynamin-related protein 1; GRP75, glucose-regulated protein 75; GRP78, glucose-regulated protein 78; VDAC, voltage-dependent anion channels; FACL4, long-chain-fatty-acid--CoA ligase; SOAT1, Sterol O-Acyltransferase 1.
