# Supplementary material for: Potential role of mitochondria and endoplasmic reticulum in the response elicited by D-aspartate in TM4 Sertoli cells
Source: Front Cell Dev Biol. 2024 Jul 22;12:1438231. doi: 10.3389/fcell.2024.1438231 (PMC11298366; doi:10.3389/fcell.2024.1438231)
Supplement: Supplementary file 1 [file Table1.DOCX]

**Table S1.** The primary antibodies used in the Western blot analysis.

| **Antibody** | **Molecular weight (kDa)** | **WB Dilution** | **Source** |
| --- | --- | --- | --- |
| Akt | 60 | 1:1000 | Cell Signaling Technology, Danvers, MA, USA  #9272 |
| AR | 110 | 1:1000 | Santa Cruz Biotechnology, Santa Cruz, CA, USA  #sc-816 |
| Bax | 23 | 1:1000 | Santa Cruz Biotechnology, Santa Cruz, CA, USA  #sc-493 |
| Bcl-2 | 25 | 1:1000 | Elabscience Biotechnology, Wuhan, China  #E-AB-60012 |
| BIP | 78 | 1:1000 | Cell Signaling Technology, Danvers, Ma, USA  #3183 |
| CATALASE | 60 | 1:1000 | Sigma-Aldrich, St. Louis, Missouri, USA  #C0979 |
| cytochrome complex | 14 | 1:1000 | Cell Signaling Technology, Danvers, Ma, USA  #4272 |
| DRP1 | 78-82 | 1:1000 | Cell Signaling Technology, Danvers, Ma, USA  #8570 |
| FACL4 | 79 | 1:1000 | Abcam, Cambridge, United Kingdom  #ab227256 |
| GRP75 | 75 | 1:1000 | Cell Signaling Technology, Danvers, Ma, USA  #2816 |
| MFN1 | 84 | 1:1000 | Abcam, Cambridge, United Kingdom  #ab221661 |
| MFN2 | 86 | 1:500 | Abcam, Cambridge, United Kingdom  #ab124773 |
| NRF1 | 68 | 1:1000 | Cell Signaling Technology, Danvers, Ma, USA  #69432 |
| p44/42 MAPK (Erk 1/2) | 42-44 | 1:1000 | Cell Signaling Technology, Danvers, Ma, USA  #9102 |
| p-Akt | 60 | 1:1000 | Cell Signaling Technology, Danvers, MA, USA  #4060 |
| PCNA | 36 | 1:1000 | Sigma-Aldrich, Milan, Italy  #98825 |
| PGC-1α | 130 | 1:1000 | Cell Signaling Technology, Danvers, MA, USA  #2178 |
| Phospho-p44/42 MAPK (Erk 1/2) | 42-44 | 1:1000 | Cell Signaling Technology, Danvers, Ma, USA  #9101 |
| SOAT1/ACAT1 | 47 | 1:1000 | Abcam, Cambridge, United Kingdom  #ab39327 |
| SOD1 | 23 | 1:1000 | Abcam, Cambridge, United Kingdom  #ab13498 |
| SOD2 | 22 | 1:4000 | Abclonal, Massachusetts, USA  #A1340 |
| Total OXPHOS | 55-48-40-30-20 | 1:1000 | Abcam, Cambridge, United Kingdom  #ab110413 |
| TFAM | 28 | 1:2000 | Abcam, Cambridge, United Kingdom  #ab131607 |
| TOMM20 | 16 | 1:1000 | Sigma-Aldrich, Milan, Italy  WH0009804M1 |
| VDAC | 32 | 1:1000 | Cell Signaling Technology, Danvers, Ma, USA  #4661 |
| β-Actin | 42 | 1:2000 | Elabscience Biotechnology, Wuhan, China  #E-AB-20031 |
